# circSIRT2/miR‐542‐3p/VASH1 axis regulates endothelial‐to‐mesenchymal transition (EndMT) in subretinal fibrosis in age‐related macular degeneration models

**DOI:** 10.1111/acel.14443

**Published:** 2025-01-02

**Authors:** Min Zhang, Jiali Wu, Yimin Wang, Yidong Wu, Xiaoling Wan, Mei Jiang, Qiyu Bo, Jieqiong Chen, Xiaodong Sun

**Affiliations:** ^1^ Department of Ophthalmology, Shanghai General Hospital Shanghai Jiao Tong University, School of Medicine Shanghai China; ^2^ National Clinical Research Center for Ophthalmic Diseases Shanghai China; ^3^ Department of Ophthalmology, Zhongshan Hospital Fudan University Shanghai China; ^4^ Shanghai Key Laboratory of Fundus Diseases Shanghai China; ^5^ Shanghai Engineering Center for Visual Science and Photomedicine Shanghai China

**Keywords:** circSIRT2, EndMT, miR‐542‐3p, subretinal fibrosis, VASH1

## Abstract

Neovascular age‐related macular degeneration (nAMD), characterized by choroidal neovascularization (CNV), is one of the leading causes of severe visual impairment and irreversible vision loss around the world. Subretinal fibrosis (SRF) contributes to the incomplete response to anti‐vascular endothelial growth factor (VEGF) treatment and is one of the main reasons for long‐term poor visual outcomes in nAMD. Reducing SRF is urgently needed in the anti‐VEGF era. The role of non‐coding RNAs has been implicated in CNV; however, their roles in SRF have not been elucidated yet. Herein, we comprehensively investigated circular RNA (circRNA) profiles in the laser‐induced mouse SRF model and the transforming growth factor‐β (TGF‐β) induced human umbilical vein endothelial cell (HUVEC) fibrosis model. A novel circRNA, circSIRT2, was identified, and its function in SRF and endothelial‐to‐mesenchymal transition (EndMT) regulation was investigated. circSIRT2 was consistently upregulated in fibrotic models in vivo and in vitro. circSIRT2 overexpression downregulated the fibrotic markers and inhibited the proliferation and migration of endothelial cells in vitro. circSIRT2 overexpression in vivo also reduced SRF area in mice. Mechanistically, circSIRT2 functioned by sponging miR‐542‐3p, which further upregulated the expression of vasohibin‐1 (VASH1) and reduced SRF lesion development. Vitreous delivery of miR‐542‐3p and VASH1 in the mouse SRF model also confirmed the pro‐fibrotic function of miR‐542‐3p and anti‐fibrotic function of VASH1, respectively. In conclusion, circSIRT2 inhibited SRF by binding miR‐542‐3p, which stimulated the VASH1 expression and subsequently suppressed EndMT. The circSIRT2/miR‐542‐3p/VASH1 axis may serve as a promising therapeutic target for SRF in nAMD.

## Graphical abstract

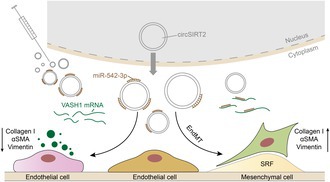

**Abbreviations**
AAV2: adeno‐associated virus‐2
ActD: actinomycin D
AMD: age‐related macular degeneration
CCK8: cell counting kit‐8
circRNA: circular RNA
CNV: choroidal neovascularization
DAPI: 4′,6‐diamidino‐2‐phenylindole
EndMT: endothelial‐to‐mesenchymal transition
FFA: fluorescein fundus angiography
FISH: fluorescence in situ hybridization
HUVECs: human umbilical vein endothelial cells
KEGG: Kyoto Encyclopedia of Genes and Genomes
lncRNA: long non‐coding RNA
miRNA: microRNA
mRNA: message RNA
nAMD: neovascular AMD
OCT: optical coherent tomography
PFA: paraformaldehyde
RNase R: ribonuclease R
RPE: retinal pigmental epithelium
RT‐qPCR: real‐time quantitative polymerase chain reaction
SEM: standard error of the mean
siRNA: small interfering RNA
SIRT2: sirtuin‐2
SRF: subretinal fibrosis
UTR: untranslated region
VASH1: vasohibin‐1
VEGF: vascular endothelial growth factor
WB: western blot
WT: wild type

## 1 INTRODUCTION

Neovascular age‐related macular degeneration (nAMD), characterized by choroidal neovascularization (CNV), is one of the leading causes of severe visual impairment and irreversible vision loss around the world (Chou et al., 2022). The anti‐vascular endothelial growth factor (VEGF) therapy has revolutionized CNV treatment, but progressive subretinal fibrosis (SRF) contributes to the incomplete response to anti‐VEGF treatment (Mettu et al., 2021). SRF is also one of the main reasons for the 10‐letter loss in eyes receiving long‐term treatment in nAMD (Gillies et al., 2020). Reducing SRF is urgently needed in the anti‐VEGF era.

SRF is generated by matrix‐generating mesenchymal cells, which can be originated from the retinal pigmental epithelium (RPE), vascular endothelium (Shu et al., 2020), and bone‐marrow‐derived macrophage (Little et al., 2020). The role of RPE in SRF via epithelial‐to‐mesenchymal transition (EMT) has been well‐illustrated (Li et al., 2021; Llorian‐Salvador et al., 2022). The involvement of endothelial‐to‐mesenchymal transition (EndMT) in fibrosis is recognized, but the understandings of its effect and regulation in fundus pathologies are limited. Cao et al. (2014) revealed the glucose‐induced EndMT in diabetic retinopathy. Long non‐coding RNAs (lncRNAs) were reported as therapeutic targets via EndMT in diabetic retinopathy (Cao et al., 2021; Thomas et al., 2019). SNAI1, an EndMT transcription factor, was reported to promote ocular neovascularization and extracellular matrix remodeling (Sun et al., 2018). However, the underlying regulating mechanism of EndMT in SRF and nAMD was still not clear.

Circular RNAs (circRNAs) are a type of endogenous non‐coding RNAs like lncRNAs. Differently, it is generated from their host genes with both ends covalently connected into a circle. Many circRNAs act as microRNA (miRNA) sponges, therefore regulating downstream message RNA (mRNA) and protein translation (Kristensen et al., 2019). The engagement of circRNAs in EndMT was realized in several systems, such as pulmonary and coronary artery tissues (Fang et al., 2018; Hulshoff et al., 2019). circRNA/miRNA/mRNA axis has been constructed for EndMT regulation in other diseases as well (Bai et al., 2018; Yang et al., 2018). Similar involvement of circRNAs and related axis could be expected in SRF regulation in nAMD.

In this study, we constructed a comprehensive circRNA profile in the laser‐induced mouse SRF model and detected the upregulation of circSirt2. We found that circSIRT2, whose host gene was sirtuin‐2 (SIRT2), significantly inhibited the EndMT by sponging miR‐542‐3p and increasing subsequent vasohibin‐1 (VASH1) expression in vivo and in vitro. Our findings elucidated the involvement of circRNA/miRNA/mRNA axis in SRF and provided a potential therapeutic target for nAMD clinical practice.

## 2 MATERIALS AND METHODS

### 2.1 Laser‐induced mouse SRF model

C57BL/6J male mice (6–8 weeks old) were purchased from the GemPharmatech laboratory animal company (Nanjing, China). Laser photocoagulation was performed as described before (Lambert et al., 2013). Briefly, after mouse anesthesia (1% sodium pentobarbital intraperitoneal injection, 50 mg/kg) and pupil dilation (0.5% tropicamide, Mydrin‐P, Santen), a 532 nm laser (Visulas 532S, Carl Zeiss) was focused on the RPE layer. Four laser spots were created in each eye between the major vessels in a relatively symmetric manner around the optic disc. Laser induction was conducted at a power of 120 mW for a duration of 100 ms. Only laser spots that produced a bubble were considered as successful Bruch's membrane disruption. Spots with hemorrhage were excluded from the subsequent study. Seven days after the laser injury, optical coherent tomography (OCT, Phoenix) and/or fluorescein fundus angiography (FFA; 2% fluorescein sodium intraperitoneal injection, Alcon; images also acquired by Phoenix) were performed to confirm the successful CNV induction. Mice were sacrificed, and eyes were enucleated 21 days post laser induction. Hematoxylin and eosin (HE), Masson's trichrome, and Sirius red staining (Service Biotech, Wuhan, China) were employed to confirm the successful induction of SRF lesions.

### 2.2 Whole transcription sequencing analysis

To profile the expression signature in SRF pathologies, the mice were sacrificed 21 days after laser induction, and the RPE‐choroid‐sclera complexes were obtained. The complexes were lysed using TRIzol reagent (Thermo Fisher Scientific). Total RNA was then extracted, followed by mRNA isolation, library construction, and sequencing by OE Biotech (Shanghai, China). Differential expression was considered statistically significant under the criterion of false discovery rate (FDR) <0.05 and absolute fold change >2.

### 2.3 Cell culture and treatment

Human umbilical vascular endothelial cells (HUVECs; CRL‐1730, ATCC) were cultured in endothelial cell medium supplemented with fetal bovine serum, endothelial cell growth supplement, and penicillin/streptomycin. The cells were maintained at 37°C with 5% CO_2_ and were passaged every 2–3 days.

Human recombinant transforming growth factor‐β (TGF‐β; 10804‐H08H, Sino Biological) were reconstituted by sterile water and stored at −20°C. The working concentration ranged from 0.1 to 10 ng/mL, and the final working concentration for EndMT induction was 10 ng/mL in cell medium.

HUVECs were transfected with Lipofectamine 3000 (Thermo Fisher Scientific) according to the manufacturer's instruction. The plasmid pcDNA3.1(+)‐S‐hsa_circ_0050951 for circSIRT2 overexpression were synthesized by OBiO Technology (Shanghai, China). VASH1 overexpression plasmid was synthesized by General Biosystem (Chuzhou, China). Small interfering RNA (siRNA) oligonucleotides for circSIRT2 and VASH1 (Table S1) and their corresponding scramble negative control, miR‐214‐3p, miR‐542‐3p, and miR‐761 mimic (micrON), as well as miR‐214‐3p and miR‐542‐3p inhibitors (micrOFF) were designed and synthesized by Ribo Biotech (Guangzhou, China). Cells were cultured for 48 hours before transfection efficiency tests and following treatment.

### 2.4 Wound healing assay

HUVECs were cultured with different treatments. When grown to 90%–100% confluency, cells were scratched with a pipette tip to create an incision‐like cross gap. The cells were washed twice in warm serum‐free medium and photographed immediately. After 8–24 h serum starvation, images were again acquired using a microscope (Nikon Eclipse Ti, Nikon Instruments). The cell migration was quantified by the percentage of scratch closures in four independent areas using Image J (Fiji, NIH, USA).

### 2.5 RNA‐fluorescence in situ hybridization (FISH) assay

circSIRT2 FISH probe mix, U6, and 18S RNA probes were synthesized by Ribo Biotech and Geneseed Biotech (Guangzhou, China). FISH assay was carried out using a FISH kit (Ribo Biotech). Briefly, HUVECs were seeded in 24‐well culture plate and fixed by 4% paraformaldehyde (PFA). The Cy3‐labeled probe was hybridized overnight. The nuclei were stained with 4′,6‐diamidino‐2‐phenylindole (DAPI; Thermo Fisher Scientific) for 10 minutes.

### 2.6 Ribonucleas R (RNase R) treatment, actinomycin D (ActD) treatment, and nucleocytoplasmic separation

For RNase R treatment, 2 μg of HUVEC total RNA was incubated at 37°C with or without 3 U/μg of RNase R (Beyotime, Shanghai, China) for 30 min. Treated RNA in two groups was reverse‐transcribed, and RT‐qPCR was performed as described below. For ActD treatment, HUVECs were treated with 5 μg/mL ActD (Sigma Aldrich) for 6–24 h to block RNA transcription. Total RNA was harvested to detect circular and linear RNA expression levels with and without ActD treatment. Nuclear and cytoplasmic fractions were isolated and extracted using NE‐PER Nuclear and Cytoplasmic Extraction Reagents (Thermo Fisher Scientific).

### 2.7 Cell counting kit‐8 (CCK8) assay

A CCK8 assay was conducted to determine the regulation effects of circSIRT2, miRNAs, and VASH1 on cell proliferation. Same number of HUVECs was seeded in 96‐well culture plates and transfected with corresponding oligonucleotides or plasmid. After 48‐h culture, CCK8 reagent (Beyotime) was added at a dilution of 1:10 for 1 h at 37°C. The number of living cells was reflected by the amount of formazan produced, which was measured by absorbance at 450 nm.

### 2.8 Real‐time quantitative polymerase chain reaction (RT‐qPCR)

RNA from HUVECs and the mouse RPE‐choroid‐sclera complexes was extracted using the RNAsimple Total RNA Kit (TianGen, Beijing, China) or the TRIzol reagent, reverse‐transcribed into cDNA using PrimeScript RT reagent Kit (TaKaRa, Dalian, China), and quantified using TB Green Premix Ex Taq (TaKaRa) on a ViiA 7 real‐time PCR system (Applied Biosystems). miRNA cDNA was synthesized using poly‐A tail extension by miRcute Plus miRNA First‐Strand cDNA Kit (TianGen) and quantified by miRcute Plus miRNA qPCR Kit with SYBR Green (TianGen). Human and mouse glyceraldehyde‐3‐phosphate dehydrogenase (GAPDH) or U6 expression were analyzed in parallel for normalization of RNA expression. Primers used are listed in Table S2.

### 2.9 Western blot (WB)

HUVECs were lysed in radio immunoprecipitation assay buffer containing protease inhibitors (Sangon Biotech, Shanghai, China) and phenylmethylsulfonyl fluoride (Beyotime). The protein was quantified by bicinchoninic acid assay (Pierce), loaded onto 10% SDS‐PAGE gels (PG112; Epizyme Biomedical, Shanghai, China), separated by electrophoresis, and transferred onto polyvinylidene fluoride membranes (Millipore). After blocking with 5% non‐fat milk (A600669, Sangon Biotech) for 1 h at room temperature, the membranes were sequentially incubated with primary antibodies and the corresponding secondary antibodies. The antibodies were as follows: anti‐GAPDH (60004‐1‐Ig, 1:1000; Proteintech), anti‐αSMA (14395‐1‐AP, 1:500; Proteintech), anti‐Collagen I (14695‐1‐AP, 1:500; Proteintech), and anti‐Vimentin (ab92547, 1:500; Abcam). Protein bands were visualized using enhanced chemiluminescence (Millipore) with a molecular imaging system (Amersham Imager 600, GE Healthcare).

### 2.10 Dual‐luciferase reporter assay

Dual‐luciferase reporter assays were conducted using the Dual Luciferase Reporter Gene Assay Kit (Beyotime) according to the manufacturer's protocol. Briefly, the wild type (WT) or mutant target sequence was cloned into the pGL6‐miR plasmid vector (firefly luciferase reporter). The WT target sequence was the 3′‐untranslated region (UTR) of mRNA, or the circRNA sequence contains the predicted binding sites. The mutant target sequences have the predicted binding sites mutated to prevent base‐pairing and binding. Sequences used in the dual‐luciferase reporter assay were listed in Table S3. The 293T cells were co‐transfected with cloned firefly plasmid, renilla plasmid, and miRNA mimics. After 48 h, the cells were collected, and firefly and renilla luciferase activity was evaluated. The renilla luciferase activity was used as an internal control of transfection efficiency. Binding or not binding was shown by the ratio of firefly luciferase activity over the renilla luciferase activity, noted as relative luciferase activity.

### 2.11 Intravitreal adeno‐associated virus 2 (AAV2) delivery

pAAV‐CMV‐S‐hsa_circ_0050951‐EF1a‐mCheTy‐3xFLAG‐WPRE AAV2 and pAAV‐CMV‐Vash1‐3xFLAG‐EF1a‐mCherry‐tWPA AAV2, as well as their corresponding vectors were constructed by OBiO Technology. The working viral titers were approximately 5 × 10^10^ vg/mL. The intravitreal injections were performed after regular mouse anesthetization and pupil dilation as described above. A 30‐gauge needle was used to create a tunnel adjacent to the limbus, and 2 μL solution of AAV2 with and without miRNA mimic/inhibitor was delivered into the intravitreal space with a 34‐gauge needle immediately after laser induction.

### 2.12 RPE‐choroid‐sclera complex flat mounting dissection, cryosection, and immunofluorescence

The RPE‐choroid‐sclera complexes or the fundus cryosections with the SRF lesion were prepared 21 days after laser injury. Mouse eyes were enucleated and fixed in 4% PFA. For the RPE‐choroid‐sclera complex preparation, the anterior segment, retina, and optic nerve were removed. The remaining eyecups were radially cut into 4 pieces from the periphery to the optic nerve head. For the cryosection preparation, tissues were dehydrated in 30% sucrose, flash‐frozen in tissue‐Tek O.C.T. compound (Sakura), and sectioned with the CM3050S Cryostat (Leica) at 12 μm thickness.

The immunofluorescence staining assay was then performed. The complexes or sections were blocked with phosphate‐buffered saline containing 0.1% Triton X‐100 and 5% bovine serum albumin for 1 h at room temperature, followed by overnight incubation with Isolectin B4 (FL‐1201, 1:500; Vector Labs), and primary antibody against α‐SMA or Collagen I. After secondary antibody incubation and DAPI staining, the flat mounts or the sections were visualized using a confocal microscope (Leica). Collage I‐positive areas on the flat amounts were considered as SRF lesion areas and quantified using Image J.

### 2.13 Statistical analyses

Statistical analyses were performed with Microsoft Excel for Mac version 16.54 (San Diego, CA, USA). Data are shown as the mean ± standard error of the mean (SEM), and the differences between two different groups were analyzed using independent sample Student's *t*‐test. *p* < 0.05 was considered as statistically significant.

## 3 RESULTS

### 3.1 Construction of the SRF models in vivo and in vitro, and validation of EndMT in the SRF lesions

In this study, the in vivo model is a mouse laser ablation model and the in vitro model is HUVECs with TGF‐β treatment.

Successful induction of CNV in mouse fundus was confirmed by OCT, FFA and color fundus images 7 days after laser burn. At the 21st day after CNV induction (CNV21d), histology staining was made (Figure S1A). HE staining indicated subretinal lesions with cells and extracellular matrix. Masson's trichrome and Sirius red stains collagen in bright blue and red, respectively. Both stains revealed significant collagen deposition in the lesions, indicating their fibrotic nature (Figure S1B, lesions indicated by arrows).

Isolectin B4 mainly binds to endothelial cells (Ernst & Christie, 2006), while αSMA is a well‐known myofibroblast marker (Shu et al., 2020). Dual staining of Isolectin B4 and αSMA in SRF lesions identified several cells, forming the cross sections of CNV (Figure S1C, boxed area with high magnification). These data suggest the involvement of EndMT in SRF lesions.

In the HUVEC EndMT model in vitro, RT‐qPCR results showed that TGF‐β treatment from 0.1 to 10 ng/mL all induced ACTA2 (αSMA) mRNA upregulation. However, consistent upregulation of fibrotic markers, that is, ACTA2, COL1A1 (Collagen I), and VIM (Vimentin) in mRNA levels, was only observed in the group of 10 ng/mL TGF‐β treatment (Figure S1D). WB also showed the increased αSMA protein level with 10 ng/mL TGF‐β treatment (Figure S1E), indicating the successful induction of EndMT in vitro. This concentration was used in the following experiments.

### 3.2 Identification of circSIRT2 in the mouse SRF model in vivo and in the HUVEC EndMT model in vitro

**FIGURE 1:**
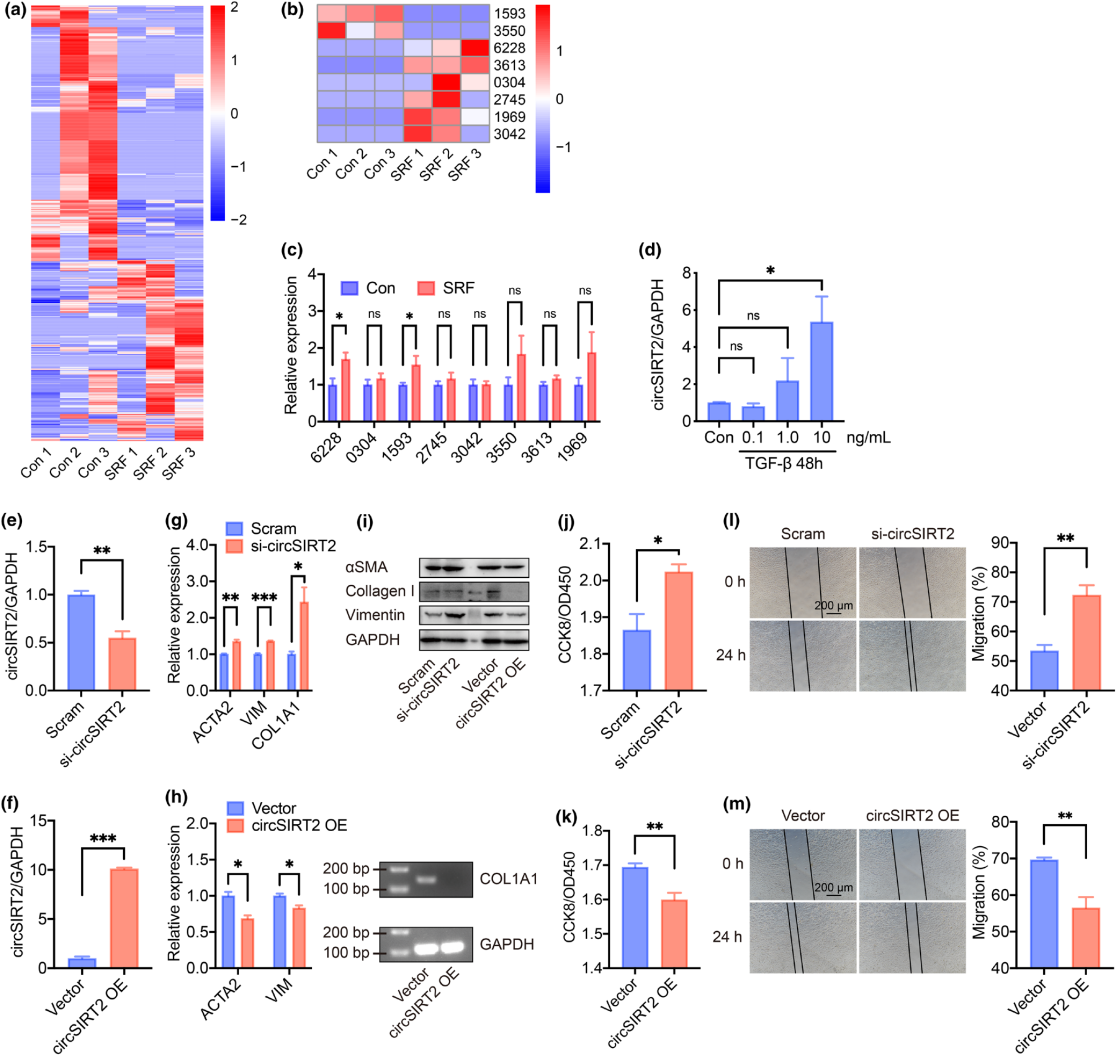
circSIRT2 was upregulated in fibrosis models in vivo and in vitro, and inhibited EndMT in HUVECs in vitro. (a) Circular RNA (circRNA) profiles of the RPE‐choroid‐sclera complexes in the mouse SRF model, visualized by R software. *n* = 3 for each group. (b) Expression signature of circRNA candidates with high fold change reported by the whole transcription sequencing analysis. *n* = 3 for each group. (c) RT‐qPCR verification of enrolled circRNAs. m_circRNA_6228 (fold change = 1.692, *p* = 0.013) and m_circRNA_1593 (fold change = 3.057, *p* = 0.045) were significantly upregulated in the mouse SRF model. As shown in (b), the whole transcription sequencing analysis reported the upregulation of m_circRNA_6228 (log_2_[fold change] = 9.210) but downregulation of m_circRNA_1593 (log_2_[fold change] = −7.735). Therefore, only m_circRNA_6228 (circSirt2) had the same regulation pattern reported by both RT‐qPCR verification and sequencing, and was chosen to be studied further. *n* = 10 for each group. (d) RT‐qPCR verification of circSIRT2 upregulation in the TGF‐β (10 ng/mL) induced HUVEC model. *n* = 3 for each group. (e) Successful knockdown of circSIRT2 in HUVECs, verified by RT‐qPCR. *n* = 4 for each group. (f) Successful overexpression of circSIRT2 in HUVECs, verified by RT‐qPCR. *n* = 3 for each group. (g) RT‐qPCR showed that downregulation of circSIRT2 induced upregulation of fibrotic markers. *n* = 3 for each group. (h) RT‐qPCR showed that upregulation of circSIRT2 induced downregulation of fibrotic markers. *n* = 3 for each group. (i) WB verified the upregulation of fibrotic markers with circSIRT2 knockdown and downregulation of fibrotic markers with circSIRT2 overexpression in the protein level. *n* = 3 for each group. (j) Increased cell proliferation with circSIRT2 knockdown was shown by CCK8 assay. *n* = 4 for each group. (k) Reduced cell proliferation with circSIRT2 overexpression was shown by CCK8 assay. *n* = 6 for each group. (l) Wound healing assay showed increased cell migration with circSIRT2 knockdown. *n* = 4 for each group. (m) Wound healing assay showed reduced cell migration with circSIRT2 overexpression. *n* = 4 for each group. ns, not significant; OE, overexpression; Scram, scramble; si‐, small interfering RNA, *p < 0.05, **p < 0.01, ***p < 0.001, independent two‐sample Student's *t*‐test. Error bars indicated SEM.

Whole‐transcriptome sequencing of the RPE‐choroid‐sclera complexes was performed to determine the circRNA profile in mouse SRF models (Figure 1a). The expression heatmap of 6 upregulated circRNAs and 2 downregulated circRNAs with high fold change in the mouse SRF model validated by RT‐qPCR was presented as Figure 1b. The mouse circRNA_6228 (Chr7:28776858_28778990_+; m_circRNA_6228) was reported to have the highest fold change (log_2_[fold change] = 9.210) in the mouse SRF model. It also had its significant increase confirmed by RT‐qPCR (fold change = 1.692, *p* = 0.013; Figure 1c) and selected to be studied further. m_circRNA_6228 was identified as mouse circRNA Sirt2 (Chr7:28776857_28778990_+; MMU_CIRCpedia_15293, circPedia, mm10; circSirt2). It has a homologue of human circRNA SIRT2 (hsa_circ_0050951, circBase, hg19; circSIRT2) with more than 90% identity (Figure S2). The upregulation of circSIRT2 was also confirmed in the 10 ng/mL TGF‐β induced EndMT model (fold change = 5.359, *p* = 0.033; Figure 1d). These data confirmed the consistent regulation of circSIRT2 in fibrosis in vivo and in vitro and suggested the potential involvement of circSIRT2 in SRF pathogenesis via EndMT.

We further investigated the circRNA nature of circSIRT2. RT‐qPCR was used to distinguish circular SIRT2 from linear SIRT2. A detailed description of circular versus linear SIRT2 primer distinction was summarized as Figure S3. First, the stability of circSIRT2 was evaluated. The ActD treatment inhibited RNA transcription. After 6 h treatment, circSIRT2 was maintained at its starting level, while linear SIRT2 was significantly degraded. The degradation of linear SIRT2 kept faster than that of circSIRT2 at each time point after 6 hours, as well (Figure S4A). RT‐qPCR results also showed that circSIRT2, but not linear SIRT2, was resistant to the RNase R digestion (Figure S4B). Moreover, the nucleocytoplasmic separation assay and RNA‐FISH assay revealed distribution of circSIRT2 in both the nucleus and cytoplasm, but was relatively more abundant in the cytoplasm (Figure S4C,D).

### 3.3 circSIRT2 inhibited EndMT in HUVECs in vitro

To determine the effect of circSIRT2 on EndMT in vitro, we constructed siRNA oligonucleotides as well as overexpression plasmid for circSIRT2 regulation and transfected them into HUVECs. Knockdown and overexpression efficiency was confirmed by RT‐qPCR (Figure 1e,f). To investigate how circSIRT2 regulate EndMT process, the expression levels of fibrotic markers were tested. ACTA2, VIM, and COL1A1 levels were found to be upregulated with circSIRT2 knockdown (Figure 1g,i) and downregulated with circSIRT2 overexpression (Figure 1h,i). As cell proliferation and migration are promoted in EndMT (Bischoff, 2019), changes in HUVEC proliferation and migration with circSIRT2 regulation were tested. The CCK8 assay (Figure 1j,k) and wound healing assay (Figure 1l,m) showed negative regulation of circSIRT2 on HUVEC proliferation and migration, respectively.

### 3.4 circSIRT2 inhibited EndMT in HUVECs by sponging miR‐542‐3p

As circSIRT2 predominantly distributed in the cytoplasm, we hypothesized that it acted as a miRNA sponge. Bioinformatic mining by circBank (http://www.circbank.cn/) reported 29 potential interacting human miRNAs. miRNA candidate with high miRNA identity between human and mouse was selected, based on the miRBase database (https://mirbase.org/). Twenty‐two human miRNAs do not have corresponding mouse miRNAs, and only three homologous miRNAs of mouse and human with 100% identity, that is, miR‐214‐3p, miR‐542‐3p, and miR‐761, were chosen to be studied further.

**FIGURE 2:**
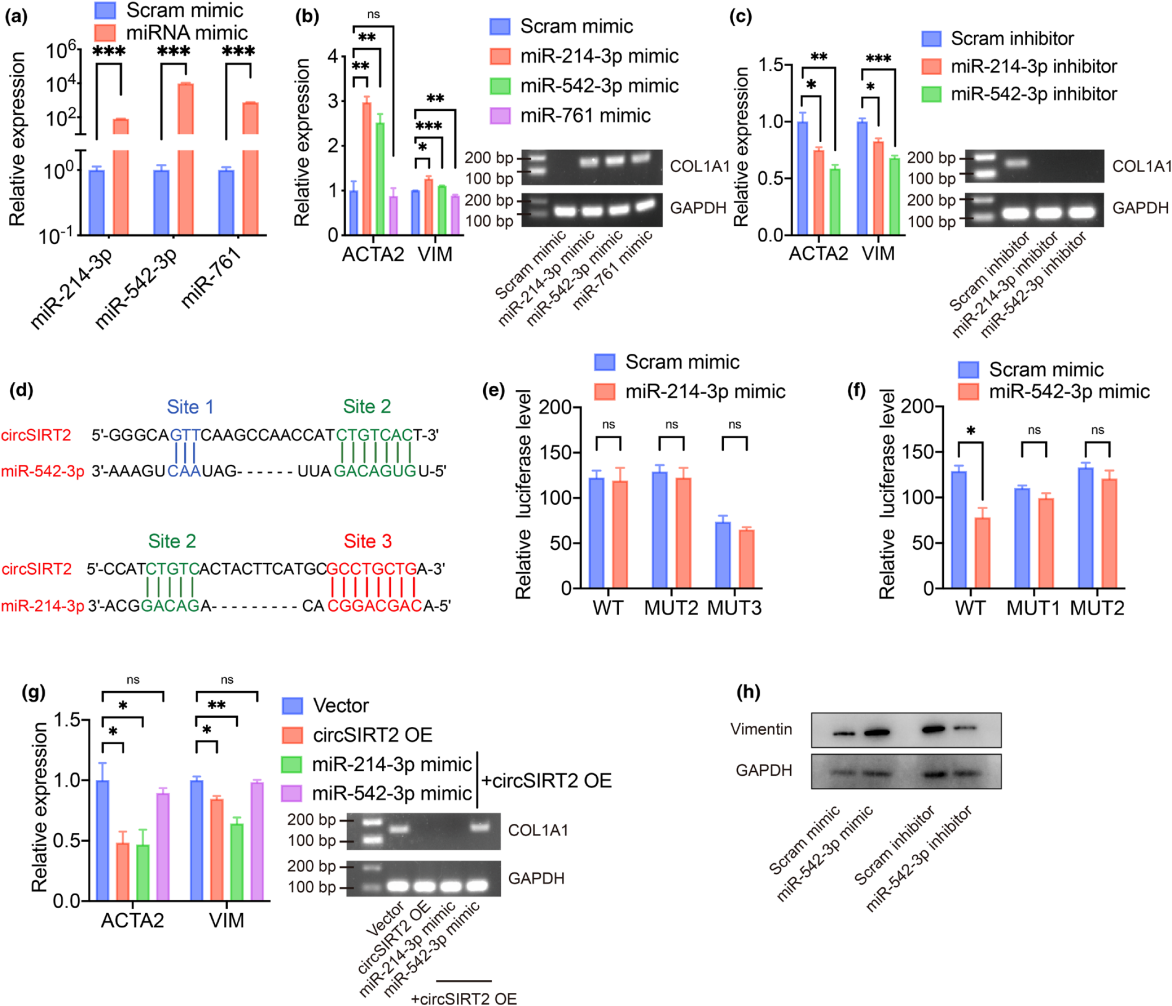
circSIRT2 regulated EndMT by sponging miR‐542‐3p. (a) Successful overexpression of miRNAs in HUVECs. *n* = 3 for each group. (b) RT‐qPCR showed that overexpression of miR‐214‐3p and miR‐542‐3p induced significant upregulation of fibrotic markers in HUVECs. *n* = 3 for each group. (c) RT‐qPCR showed that inhibition of miR‐214‐3p and miR‐542‐3p induced significant downregulation of fibrotic markers in HUVECs. *n* = 3 for each group. (d) The predicted binding sites of miRNAs and circSIRT2. (e) LUC‐circSIRT2‐WT, LUC‐circSIRT2‐MUT2, or LUC‐circSIRT2‐MUT3 (corresponding to the mutant binding sites 2 and 3, respectively), and renilla luciferase plasmid, with miR‐214‐3p mimic or scramble mimic were co‐transfected into 293T cells. Luciferase activity was detected 48 h after transfection. The renilla luciferase activity was used as an internal control of transfection efficiency. No reduction was found in relative luciferase activity of LUC‐circSIRT2‐WT with miR‐214‐3p, indicating no bindings of circSIRT2 and miR‐214‐3p. *n* = 3 for each group. (f) LUC‐circSIRT2‐WT, LUC‐circSIRT2‐MUT1, or LUC‐circSIRT2‐MUT2 (corresponding to the mutant binding sites 1 and 2, respectively), and renilla luciferase plasmid, with miR‐542‐3p mimic or scramble mimic were co‐transfected into 293T cells. Reduced relative luciferase activity of LUC‐circSIRT2‐WT with miR‐542‐3p indicated bindings of circSIRT2 and miR‐542‐3p. Similar relative luciferase activity of LUC‐circSIRT2‐MUT1 or LUC‐circSIRT2‐MUT2 with miR‐542‐3p and with scramble mimic indicated no bindings of mutant circSIRT2 sequences and miR‐542‐3p. *n* = 3 for each group. (g) RT‐qPCR showed that miR‐542‐3p overexpression could reverse the downregulation of fibrotic markers introduced by circSIRT2 overexpression. *n* = 3 for each group. (h) WB verified the upregulation of Vimentin with miR‐542‐3p overexpression (fold change = 1.700, *p* = 0.014, *n* = 6) and downregulation of Vimentin with miR‐542‐3p inhibition (fold change = 0.701, *p* = 0.027, *n* = 4) in the protein level. *p* Values reported by one‐sample Student's *t*‐test, null hypothesis mean = 1. ns, not significant; OE, overexpression; Scram, scramble, *p < 0.05, **p < 0.01, ***p < 0.001, independent two‐sample Student's *t*‐test, if not indicated. Error bars indicated SEM.

Corresponding miRNA mimics were transfected into HUVECs and successfully increased their expression (Figure 2a). Overexpression of miR‐214‐3p and miR‐542‐3p induced significant upregulation of fibrotic markers in HUVECs, and vice versa (Figure 2b,c). These results indicated that these two miRNAs exerted a pro‐EndMT function in vitro. We then constructed firefly luciferase reporters by cloning the binding sites for both miR‐214‐3p and miR‐542‐3p (Figure 4d), and the dual‐luciferase reporter assay was performed. The relative luciferase activity was only reduced in the group of circSIRT2 sequence (LUC‐circSIRT2‐WT) with miR‐542‐3p mimic, but not with miR‐214‐3p mimic (Figure 2e,f). In addition, mutating the predicted binding sites of circSIRT2 (LUC‐circSIRT2‐MUT1 and LUC‐circSIRT2‐MUT2) neutralized the reduced relative luciferase activity, indicating the abolished sponging effect between them. In addition, supplementing miR‐542‐3p but not miR‐214‐3p successfully reversed the anti‐fibrotic effect of circSIRT2 in HUVECs (Figure 2g). WB also verified the positive regulation of Vimentin with miR‐542‐3p in the protein level (Figure 2h).

**FIGURE 3:**
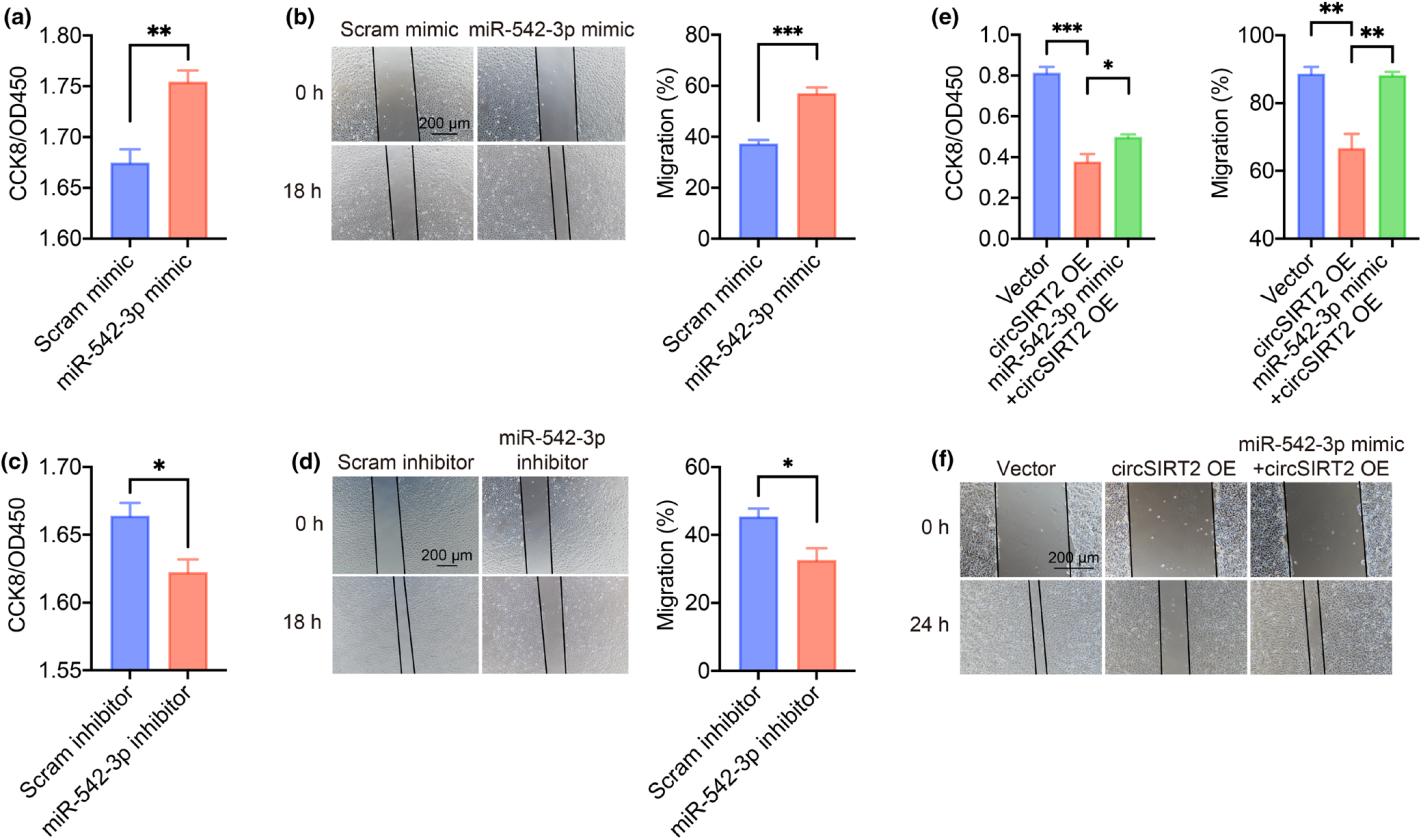
miR‐542‐3p promoted proliferation and migration of HUVECs. (a) Increased cell proliferation with miR‐542‐3p mimic was shown by CCK8 assay. *n* = 6 for each group. (b) Wound healing assay showed increased cell migration with miR‐542‐3p mimic. *n* = 4 for each group. (c) Reduced cell proliferation with miR‐542‐3p inhibition was shown by CCK8 assay. *n* = 6 for each group. (d) Wound healing assay showed reduced cell migration with miR‐542‐3p inhibition. *n* = 4 for each group. (e) CCK8 assay showed that reduced cell proliferation with circSIRT2 overexpression was reversed by miR‐542‐3p mimic. *n* = 3 for each group. (f) Wound healing assay showed reduced cell migration with circSIRT2 overexpression was reversed by miR‐542‐3p mimic. *n* = 4 for each group. ns, not significant; OE, overexpression; Scram, scramble, *p < 0.05, **p < 0.01, ***p < 0.001, independent two‐sample Student's *t*‐test. Error bars indicated SEM.

We tested the cell proliferation and migration with miR‐542‐3p regulation, as well. Increased HUVEC proliferation and migration were observed with miR‐542‐3p mimic compared to those with scramble mimic (Figure 3a,b), and reduced proliferation and migration with miR‐542‐3p inhibitor (Figure 3c,d). The overexpression of miR‐542‐3p also fully or partially abrogated the reduced cell proliferation and migration brought by circSIRT2 overexpression (Figure 3e,f). These results confirmed the involvement of miR‐542‐3p in EndMT and the downstream position of miR‐542‐3p in circSIRT2 regulation.

### 3.5 miR‐542‐3p targeted VASH1

**FIGURE 4:**
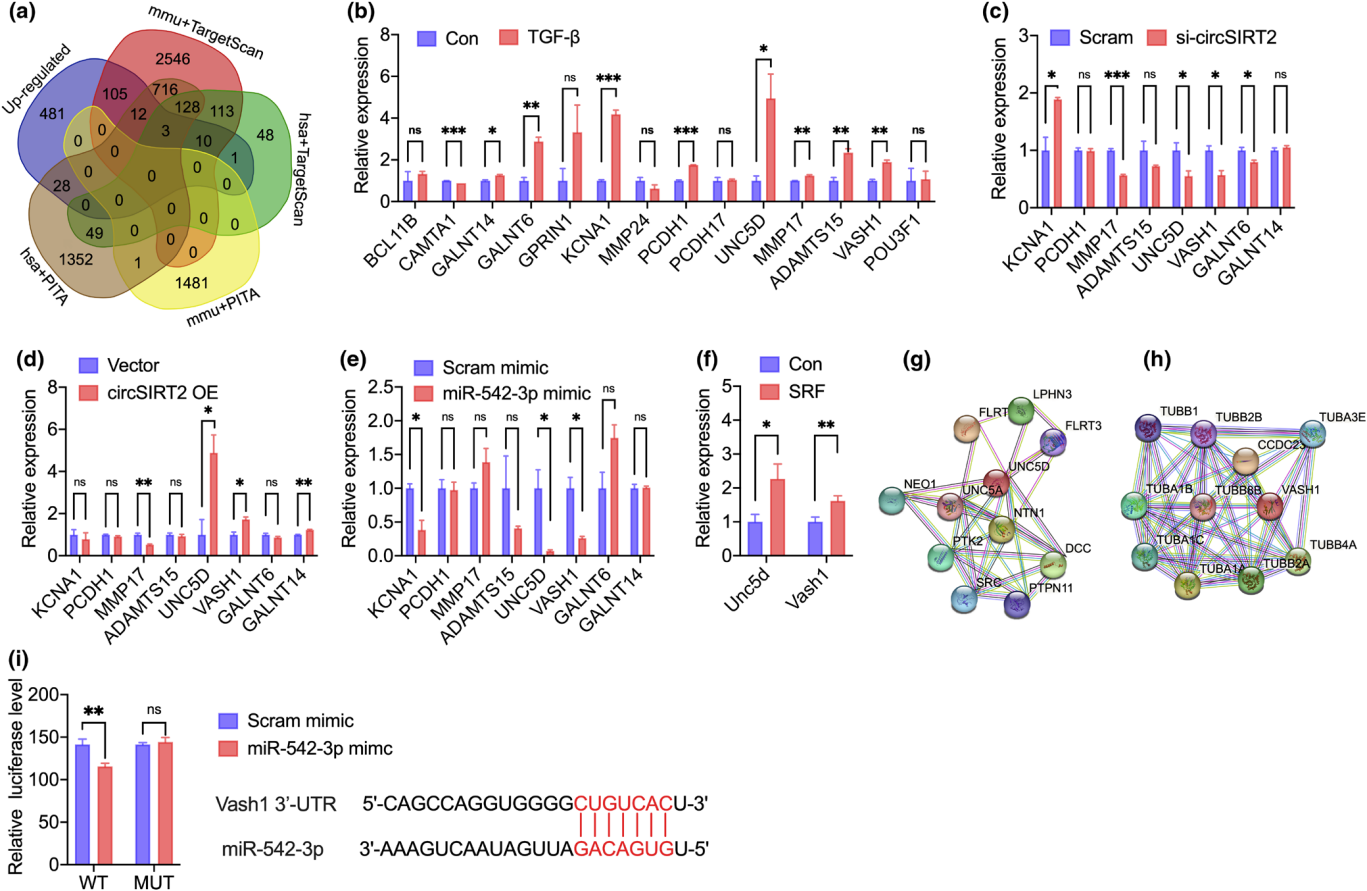
Bioinformatic prediction and selection of miR‐542‐3p target genes. (a) Venn diagram presented miR‐542‐3p target gene candidates predicted by RNA sequencing and public database. Upregulated genes were merged with bioinformatic analyses based on hsa‐miR‐542‐3p and mmu‐miR‐542‐3p in public database TargetScan (https://www.targetscan.org/) and PITA (https://tools4mirs.org/). “mmu” indicated prediction based on mouse reference genome; “hsa” indicated predication based on human reference genome. (b) RT‐qPCR validation of potential target genes in the TGF‐β induced HUVEC EndMT model. KCNA1, PCDH1, MMP17, ADAMTS15, UNC5D, VASH1, GALNT6, and GALNT14 were significantly upregulated in vitro. *n* = 3 for each group. (c–e) RT‐qPCR validation of potential target genes in HUVECs with circSIRT2 downregulated, circSIRT2 overexpressed, and miR‐542‐3p overexpressed, respectively. Only UNC5D and VASH1 were consistently downregulated with circSIRT2 downregulation and miR‐542‐3p overexpression, and upregulated with circSIRT2 overexpression, following the rule of circSIRT2/miR‐542‐3p/target gene regulation. *n* = 3 for each group. (f) RT‐qPCR verified the upregulation of Unc5d and Vash1 in the mouse SRF model. *n* = 10 for each group. (g, h) Protein–protein interactions centered on UNC5D and VASH1, respectively, predicted and visualized by STRING (https://cn.string‐db.org/). (i) The predicted binding site of Vash1 mRNA 3′‐UTR and miR‐542‐3p indicated in red. LUC‐Vash1‐WT or LUC‐Vash1‐MUT, and renilla luciferase plasmid, with miR‐542‐3p mimic or scramble mimic were co‐transfected into 293T cells. Luciferase activity was detected 48 h after transfection. Reduced relative luciferase activity of LUC‐Vash1‐WT with miR‐542‐3p indicated bindings of Vash1 mRNA 3′‐UTR and miR‐542‐3p. Similar relative luciferase activity of LUC‐Vash1‐MUT with miR‐542‐3p indicated no bindings of mutant Vash1 mRNA 3′‐UTR sequences and miR‐542‐3p. *n* = 6 for each group. Con, control; ns, not significant; OE, overexpression; Scram, scramble; si‐, small interfering RNA; SRF, subretinal fibrosis, *p < 0.05, **p < 0.01, ***p < 0.001, independent two‐sample Student's *t*‐test. Error bars indicated SEM.

To fully analyze the effect of miR‐542‐3p on gene differential expression, we conducted RNA sequencing to determine the transcription profile in the mouse SRF model (Figure S5A). A total of 640 significantly upregulated gene were revealed. Bioinformatic analysis based on hsa‐miR‐542‐3p and mmu‐miR‐542‐3p in public database TargetScan (https://www.targetscan.org/) and PITA (https://tools4mirs.org/) also identified thousands of potential target genes (Figure 4a). The 14 merged genes with high fold change in sequencing (Figure S5B,C) were tested by RT‐qPCR. Genes verified to be significantly upregulated with TGF‐β treatment (Figure 4b) were then tested in HUVECs with circSIRT2 and miR‐542‐3p regulation (Figure 4c–e). Only UNC5D and VASH1 were upregulated with circSIRT2 overexpression and downregulated with circSIRT2 knockdown and miR‐542‐3p overexpression. Both genes were upregulated in the mouse SRF model, as well (Figure 4f).

We further studied the function of UNC5D and VASH1 by literature review and identified their own predicted functional partners. The STRING database (https://cn.string‐db.org/) was used to predict and visualize the protein–protein interactions centered on these two proteins (Figure 4g,h). UNC5D mediates nerve growth (Zhu et al., 2013). Its upstream Netrin‐1 may contribute to pathological vascular permeability (Figure 4g), but mainly via another receptor UNC5B (Boye et al., 2022; Miloudi et al., 2016). In addition, though we verified significant changes in UNC5D in HUVECs with different treatment, its absolute expression levels were quite low, which was also indicated by Yang et al (Yang et al., 2007). Immunostaining of Unc5d were not co‐identified with Isolectin B4 in mouse retina (Liu et al., 2014), indicating its limited expression level in eye vascular endothelium, either. Meanwhile, VASH1 interacts with tubulin family members (Figure 4h), mediating the microtubules re‐organization and inhibits angiogenesis in many pathologies (Aillaud et al., 2017; Kobayashi et al., 2021). Compared to UNC5D, VASH1 was expected to be more involved in endothelial cell pathologies and was selected to be miR‐542‐3p target gene in the following analyses.

The binding sites for miR‐542‐3p in the Vash1 mRNA 3′‐UTR was predicted using the TargetScan database (Figure 4i). Dual‐luciferase reporter assay showed that miR‐542‐3p decreased the relative luciferase activity with the Vash1 mRNA 3′‐UTR (LUC‐Vash1‐WT). The reduction was attenuated with the mutant binding site (LUC‐Vash1‐MUT). The base‐pairing interaction of miR‐542‐3p with Vash1 was verified.

### 3.6 VASH1 inhibited EndMT in HUVECs and circSIRT2/miR‐542‐3p/VASH1 axis regulated endothelial function

**FIGURE 5:**
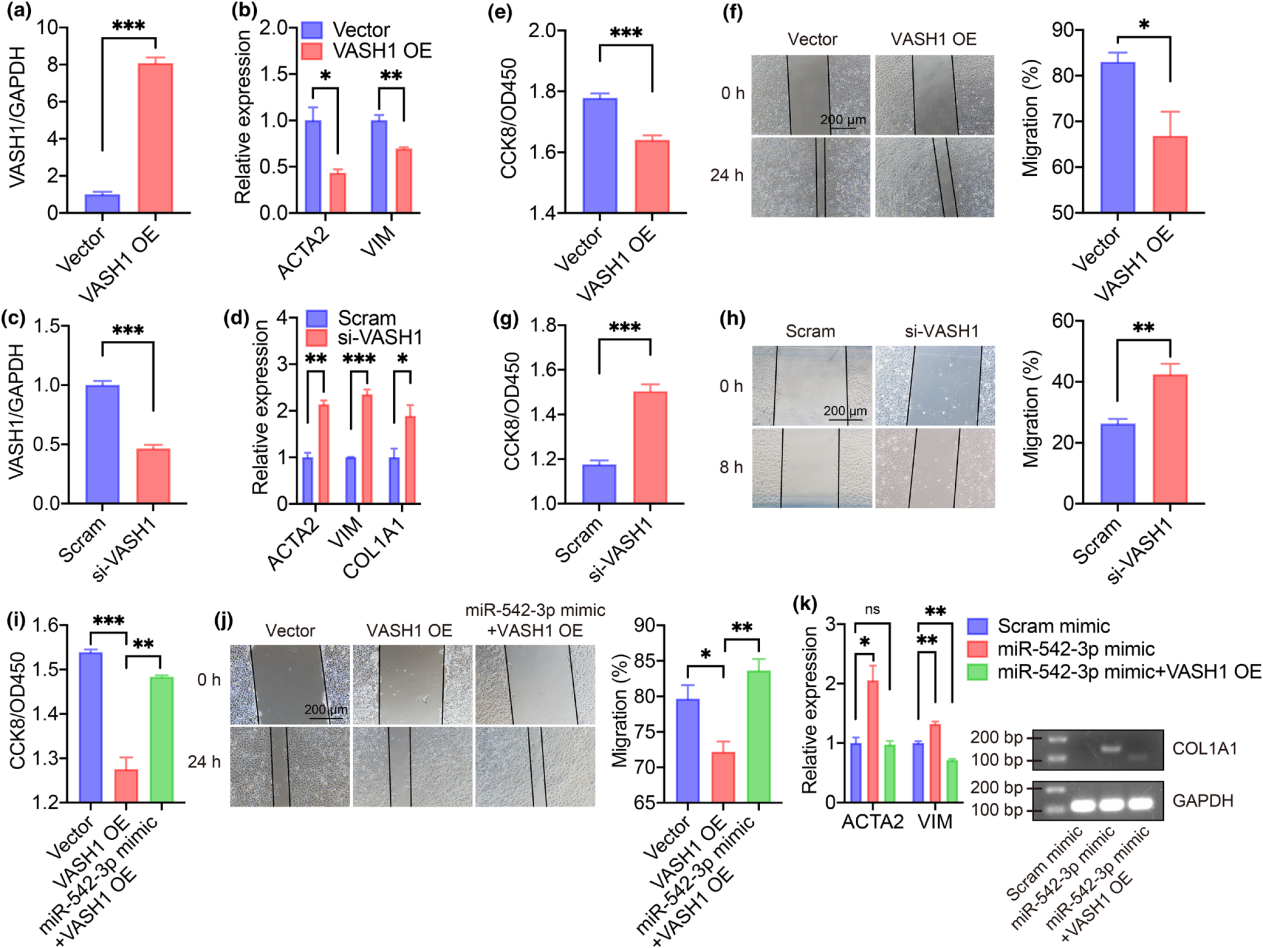
VASH1 inhibited EndMT in vitro. (a) RT‐qPCR showed successful overexpression of VASH1 in HUVECs. *n* = 3 for each group. (b) RT‐qPCR showed that VASH1 overexpression induced downregulation of fibrotic markers. *n* = 3 for each group. (c) RT‐qPCR showed that si‐VASH1 oligonucleotides successfully downregulated VASH1 in HUVECs. *n* = 3 for each group. (d) RT‐qPCR showed that VASH1 downregulation induced upregulation of fibrotic markers. *n* = 3 for each group. (e) Reduced cell proliferation with VASH1 overexpression was shown by CCK8 assay. *n* = 6 for each group. (f) Wound healing assay showed reduced cell migration with VASH1 overexpression. *n* = 4 for each group. (g) Increased cell proliferation with VASH1 downregulation was shown by CCK8 assay. *n* = 5 for each group. (h) Wound healing assay showed increased cell migration with VASH1 downregulation. *n* = 4 for each group. (i) CCK8 assay showed that additional miR‐542‐3p mimic abrogated cell proliferation reduction with VASH1 overexpression. *n* = 3 for each group. (j) Wound healing assay showed that additional miR‐542‐3p abrogated cell migration reduction with VASH1 overexpression. *n* = 4 for each group. (k) RT‐qPCR results showed that additional VASH1 overexpression abrogated pro‐fibrotic effect of miR‐542‐3p mimic. *n* = 3 for each group. ns, not significant; OE, overexpression; Scram, scramble; si‐, small interfering RNA, *p < 0.05, **p < 0.01, ***p < 0.001, independent two‐sample Student's *t*‐test. Error bars indicated SEM.

To elucidate the function of VASH1 in EndMT, we overexpressed VASH1 in HUVECs (Figure 5a). The expression of ACTA2, COL1A1, and VIM was downregulated with VASH1 overexpression (Figure 5b). The proliferation and migration of HUVECs were also reduced with VASH1 overexpression (Figure 5e,f). Opposite biological effects were achieved with VASH1 knockdown in HUVECs (Figure 5c,d,g,h). These results indicated the anti‐EndMT effects of VASH1 in vitro. Moreover, the overexpression of miR‐542‐3p abrogated the proliferation and migration reduction brought by VASH1 (Figure 5i,j). VASH1 also downregulated the fibrotic markers upregulated by miR‐542‐3p mimic (Figure 5k). Therefore, we concluded that VASH1 inhibited EndMT in HUVECs and was the downstream of miR‐542‐3p. circSIRT2/miR‐542‐3p/VASH1 axis regulated in endothelial EndMT in vitro.

### 3.7 circSIRT2/miR‐542‐3p/VASH1 axis regulated SRF in the mouse SRF model in vivo

**FIGURE 6:**
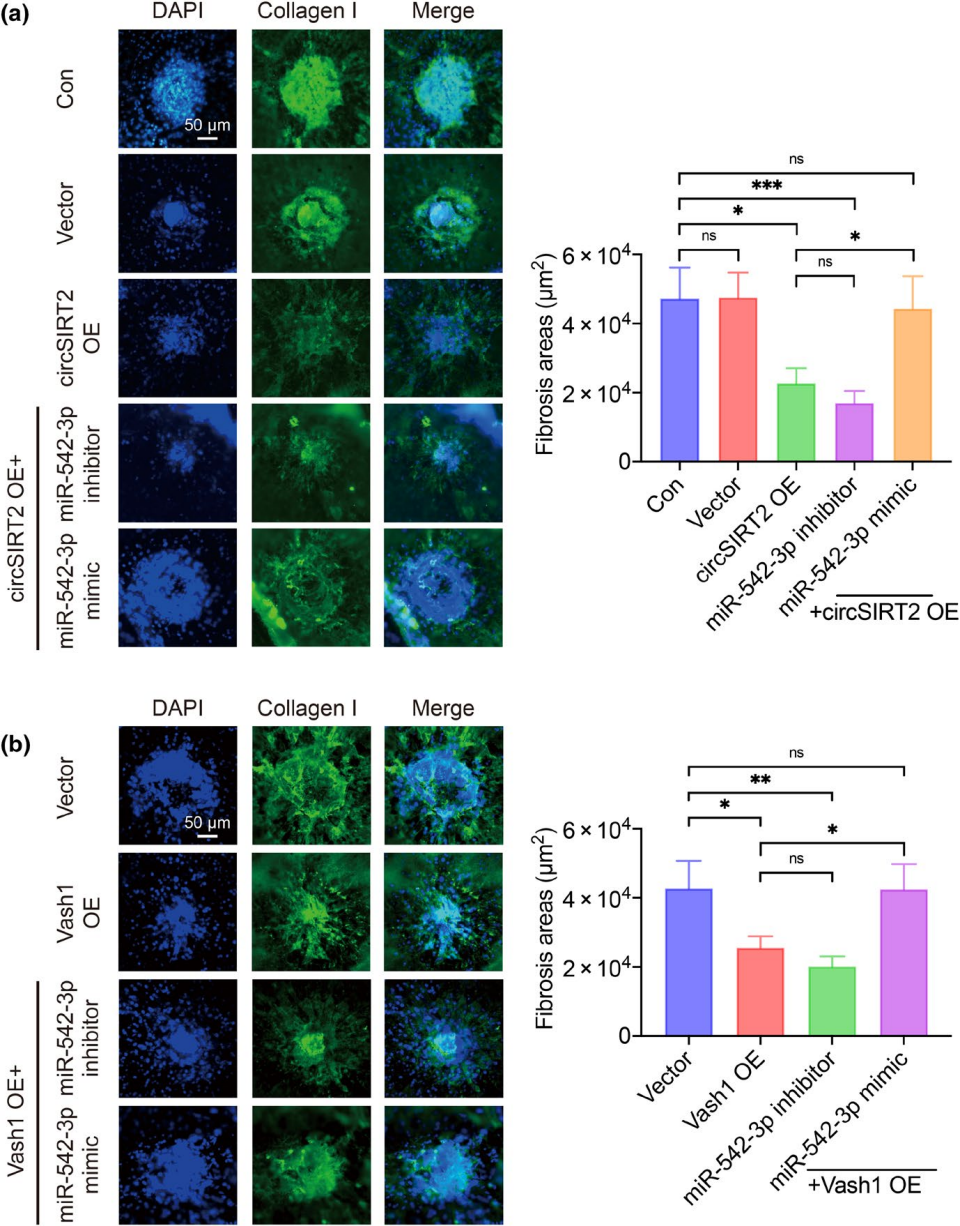
circSIRT2/miR‐542‐3p/VASH1 axis regulated SRF in vivo. (a) The intravitreal delivery of circSIRT2 overexpression AAV2 reduced the SRF lesion areas in vivo. The overexpression of miR‐542‐3p abrogated the anti‐fibrotic effect brought by circSIRT2, while miR‐542‐3p inhibition not. *n* = 7, 20, 17, 16, and 17 for the control, vector, circSIRT2 overexpression, circSIRT2 overexpression with miR‐542‐3p inhibitor, and circSIRT2 overexpression with miR‐542‐3p mimic group, respectively. (b) The intravitreal delivery of Vash1 overexpression AAV2 reduced the SRF lesion areas in vivo. The overexpression of miR‐542‐3p abrogated the anti‐fibrotic effect brought by Vash1, while miR‐542‐3p inhibition not. *n* = 13, 19, 16, and 17 for the vector, Vash1 overexpression, Vash1 overexpression with miR‐542‐3p inhibitor, and Vash1 overexpression with miR‐542‐3p mimic group, respectively. Con, control; OE, overexpression, ns = not significant, *p < 0.05, **p < 0.01, ***p < 0.001, reported by independent two‐sample Student's *t*‐test. Error bars indicated SEM.

To reveal the function of circSIRT2/miR‐542‐3p/VASH1 axis in SRF in vivo, circSIRT2 or Vash1 overexpression AAV2 with and without miR‐542‐3p mimic/inhibitor were delivered into the mouse vitreous body immediately after laser induction. We isolated the RPE‐choroid‐sclera complexes to generate the flat amounts and stained them with Collagen I antibody to evaluate the SRF area. The quantitative results showed that both circSIRT2 (Figure 6a) and Vash1 (Figure 6b) exerted the anti‐fibrosis effects, whereas miR‐542‐3p promoted SRF enlargement. These results were consistent with those findings in vitro, confirming the regulation effects of circSIRT2/miR‐542‐3p/VASH1 axis in SRF via EndMT.

## 4 DISCUSSION

In this study, we demonstrated the upregulation of circSIRT2 in the laser‐induced mouse SRF model, and its role in inhibiting EndMT by sponging miR‐542‐3p and boosting VASH1 expression. After supplementing circSIRT2 in vitro and in vivo, EndMT were suppressed in HUVECs and the SRF areas were significantly reduced in mice. Our results demonstrated the potentials of circSIRT2/miR‐542‐3p/VASH1 axis as a target for SRF prevention and treatment.

Endothelial cells are the major target of pathological alterations in nAMD, from neovascularization to SRF. Previously, we revealed circUxs1/miR‐335‐5p/PGF axis as the regulator of CNV pathology by targeting endothelial cells (Wu et al., 2023). In another study, we showed that dual inhibition of integrin α5β1 and VEGF had joint effects in attenuating angiogenesis via inhibiting endothelial cell migration, tube formation, and proliferation (Wang et al., 2016). Endothelial cells may also contribute to the accumulation of fibroblasts via EndMT, which is one of the main effector cells of fibrosis (Bischoff, 2019). For example, Zou et al. (2022) reported that Yes‐associated protein promotes EndMT in CNV fibrosis. In this study, we identified fibrotic markers in CNV vascular cells. We also found that circSIRT2 attenuated SRF area in vivo and rescued HUVECs from transiting into mesenchymal cells in vitro, indicating the involvement of EndMT in SRF pathologies and the significant role of circSIRT2 in this process.

circSIRT2 is primarily derived from precursor SIRT2 mRNA and should generate from back‐splicing mechanism. Though, to our best knowledge, circSIRT2 has not been studied before, the role of other circRNAs in different phases of fibrosis has been elucidated in other tissues or systems, including inflammation, regulation of effector cells, and the regulation of extracellular matrix (Yang et al., 2021). For example, circFBXW7 prevents the intensification of inflammation and damage of hepatic fibrosis by upregulating proinflammatory cytokines TNF‐α and IL‐1β and downregulating anti‐inflammatory cytokine IL‐10 (Chen et al., 2020); circHIPK3 felicitates the expression of pro‐fibrotic genes SOX4 and COL1A1, and promotes fibroblast activation and pulmonary fibrosis (Zhang et al., 2019); circAKT3 and circRNA_010383 have the similar role in inhibiting extracellular matrix accumulation in diabetic nephropathy (Peng et al., 2021; Tang et al., 2020). In this study, we found that circSIRT2 inhibited EndMT in vitro and reduced the SRF area in vivo. Our results identified a new circRNA, circSIRT2, in the fibrosis pathology and added information of circRNAs to SRF and nAMD.

We further identified miR‐542‐3p and VASH1 as downstream regulators in the EndMT and SRF. miR‐542‐3p is found to drive renal fibrosis by targeting AGO1 (Li et al., 2020), control hepatic stellate cell activation and fibrosis by targeting BMP7 (Ji et al., 2019), and contribute to dermal fibroblast apoptosis in systematic sclerosis by targeting Survivin (Vahidi Manesh et al., 2019). Herein, we also detected the pro‐fibrotic effect of miR‐542‐3p in eye fundus. We then identified its negative effects on VASH1 regulation in EndMT. VASH1 encodes vasohibin‐1, which regulates tubulin detyrosination, actin binding, and metallocarboxypeptidase activity (Liao et al., 2019; Nieuwenhuis et al., 2017). It is also reported to prevent pulmonary fibrosis (Fukui et al., 2021; Qu et al., 2016), hepatic fibrosis (Chatterjee, 2014; Coch et al., 2014), and fibrosis in diabetic kidney disease (Hinamoto et al., 2014; Ren et al., 2020). Consistently, in this study, we confirmed the anti‐fibrotic role of VASH1 in SRF. Therefore, the circSIRT2/miR‐542‐3p/VASH1 axis was constructed and its role in EndMT and SRF was clarified.

There were some limitations in this study, such as no validation of circSIRT2/miR‐542‐3p/VASH1 levels in human samples. The functions of circSIRT2 were not fully addressed, either. This study focused on circSIRT2 function in cytoplasm as a miRNA sponge. As circSIRT2 is distributed in both the nucleus and cytoplasm, it might interact with RNA‐binding proteins in nucleus as well. In addition, its effects on RPE, pericytes, and/or other cells in the subretinal matrix were not studied. More investigations on the roles of circSIRT2 in RPE cells and EMT, or other types of cells able to transit into mesenchymal cells, are needed.

## 5 CONCLUSIONS

circSIRT2 inhibited SRF by binding miR‐542‐3p, which stimulated the VASH1 expression and subsequently suppressed EndMT. The circSIRT2/miR‐542‐3p/VASH1 axis may serve as a promising therapeutic target for SRF in nAMD.

## AUTHOR CONTRIBUTIONS

All authors contributed to the study conception and design. Material preparation, data collection, and analysis were performed by Min Zhang, Jiali Wu, Yimin Wang, Yidong Wu, Qiyu Bo, and Jieqiong Chen. Project was administrated by Xiaodong Sun, Mei Jiang, and Xiaoling Wan. The first draft of the manuscript was written by Min Zhang, and all authors commented on previous versions of the manuscript. All authors read and approved the final manuscript.

## FUNDING INFORMATION

This study was supported by the National Natural Science Foundation of China (82301221, 82171076, 82301220, 82101159) and Shanghai Sailing Program (22YF1435500, 23YF1434100).

## CONFLICT OF INTEREST STATEMENT

Not applicable.

## CONSENT TO PARTICIPATE AND CONSENT TO PUBLISH

Human participants were not involved, neither their data nor biological materials. Consent to participate or consent to publish was not applicable.

## Supporting information


Data S1

## Data Availability

The data that support the findings of this study are available from the corresponding author upon reasonable request.
