# Supplementary material for: circSIRT2/miR‐542‐3p/VASH1 axis regulates endothelial‐to‐mesenchymal transition (EndMT) in subretinal fibrosis in age‐related macular degeneration models
Source: Aging Cell. 2025 Jan 2;24(4):e14443. doi: 10.1111/acel.14443 (PMC11984685; doi:10.1111/acel.14443)
Supplement: Supplementary file 1 — Data S1 [file ACEL-24-e14443-s001.docx]

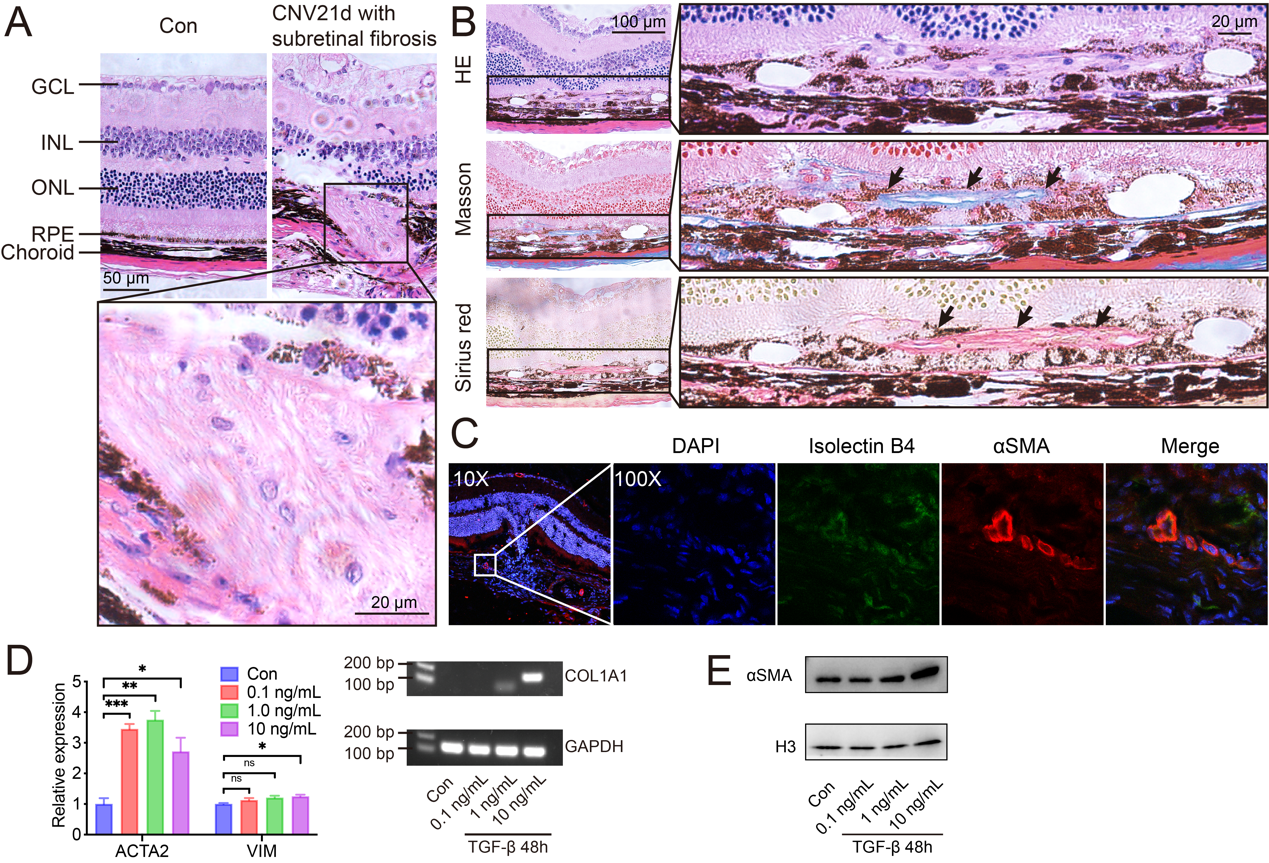


Supplementary Figure S1. Construction and validation of fibrosis and EndMT models *in vivo* and *in vitro*.

1. Successful induction of subretinal fibrosis (SRF) *in vivo* at 21 days post choroidal neovascularization induction (CNV21d) in mice, stained by hematoxylin and eosin (HE) staining. Boxed area with high magnification presented a SRF lesion. GCL = ganglion cell layer, INL = inner nuclear layer, ONL = outer nuclear layer, RPE = retinal pigmental epithelium.
2. HE, Masson's trichrome, and Sirius red staining of SRF lesions. Arrows highlighted the significant collagen deposition in the lesions, stained in bright blue by Masson and in red by Sirius red, respectively.
3. Immunofluorescence images of a SRF lesion. Dual staining of Isolectin B4 and αSMA was observed, indicating the involvement of endothelial-to-mesenchymal transition (EndMT) in the SRF lesion.
4. Successful induction of EndMT *in vitro* in human umbilical vein endothelial cells (HUVECs) with TGF-β treatment. RT-qPCR results showed consistent upregulation of fibrotic markers, i.e., ACTA2 (αSMA), COL1A1 (Collagen I), and VIM (Vimentin) in HUVECs with 10 ng/mL TGF-β treatment in the mRNA level. n = 3 for each group. ns = not significant, ^*^*P* <0.05, ^**^*P* <0.01, ^***^*P* <0.001, independent two-sample student’s *t* test. Error bars indicated SEM.
5. WB showed upregulation of αSMA in HUVECs with 10 ng/mL TGF-β treatment.


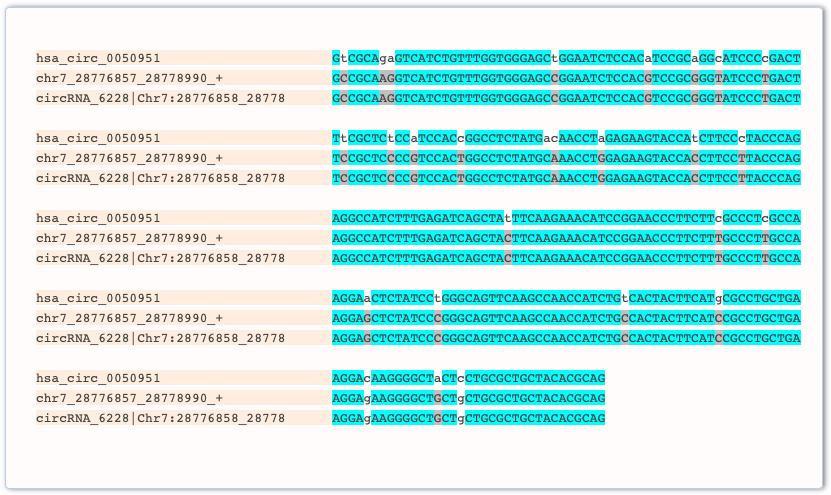


Supplementary Figure S2. Alignment of human circSIRT2 (hsa_circ_0050951), mouse circSirt2 (Chr7:28776857_28778990_+; MMU_CIRCpedia_15293) and mouse circRNA_6228 (Chr7:28776858_28778990_+) sequences. Generated by NovoPro Multiple Sequence Comparison online tool (https://www.novopro.cn/tools/muscle.htm).


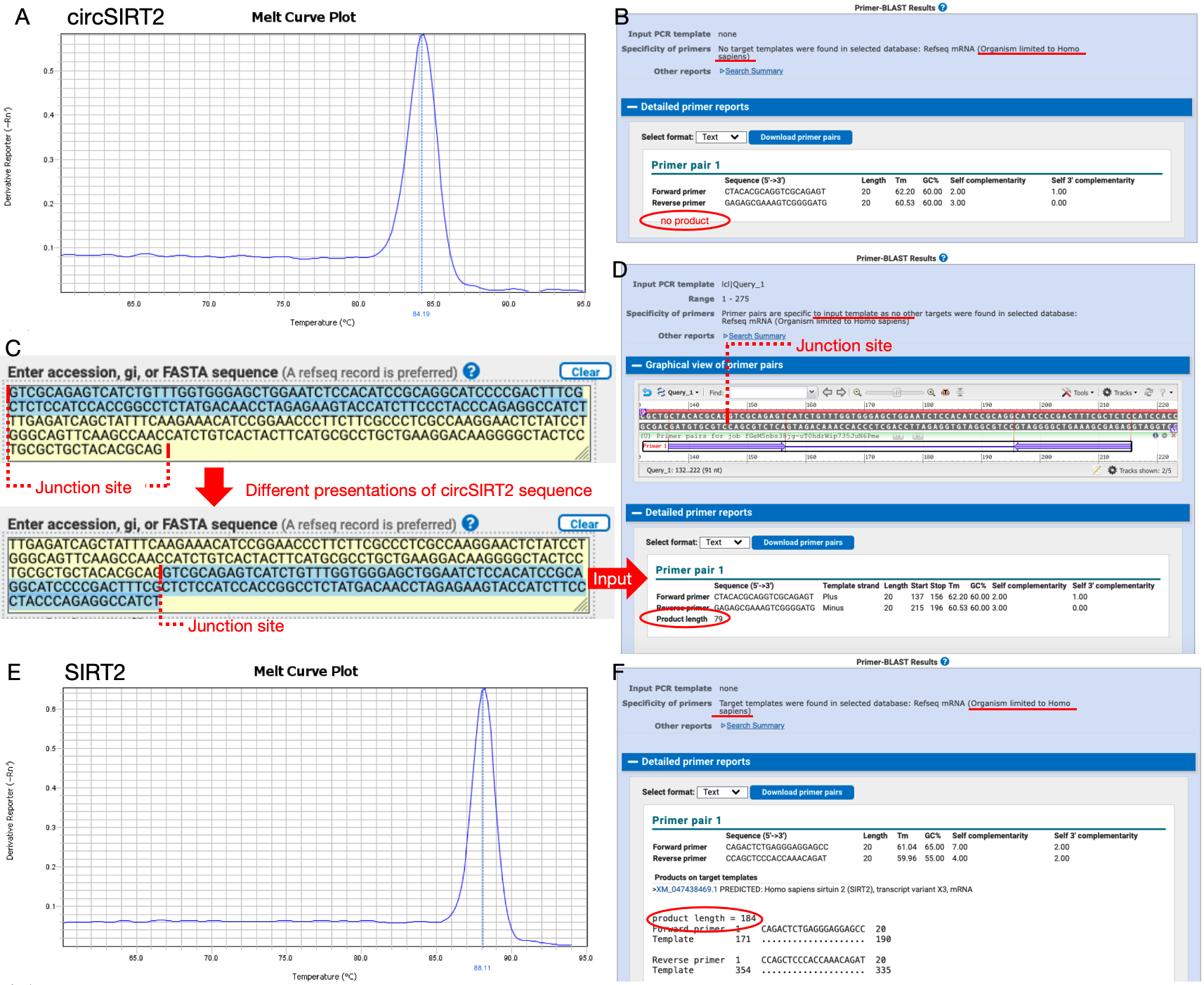


Supplementary Figure S3. Distinctions between circular SIRT2 (circSIRT2) and linear SIRT2 (SIRT2) by RT-qPCR and validation of their primers.

1. Melting curve plot of circSIRT2 RT-qPCR product. Its melting temperature was ~84℃.
2. Blast of circSIRT2 primers. Organism limited to *Homo sapiens*, but no target was found. Report generated by Primer-BLAST (https://www.ncbi.nlm.nih.gov/tools/primer-blast/).
3. The circSIRT2 sequence was reorganized to have its junction site in the middle of the sequence rather than at the ends of the sequence.
4. The primers were blasted with the reorganized circSIRT2 sequence using Primer-BLAST Results showed that the product size is 79 base pairs (bp). The forward primer spanned the circSIRT2 junction site.
5. Melting curve plot of linear SIRT2 RT-qPCR product. Its melting temperature was ~88℃.
6. Blast of linear SIRT2 primers. Organism limited to *Homo sapiens*, SIRT2 mRNA was found to be the target template. The product size was 184 bp.


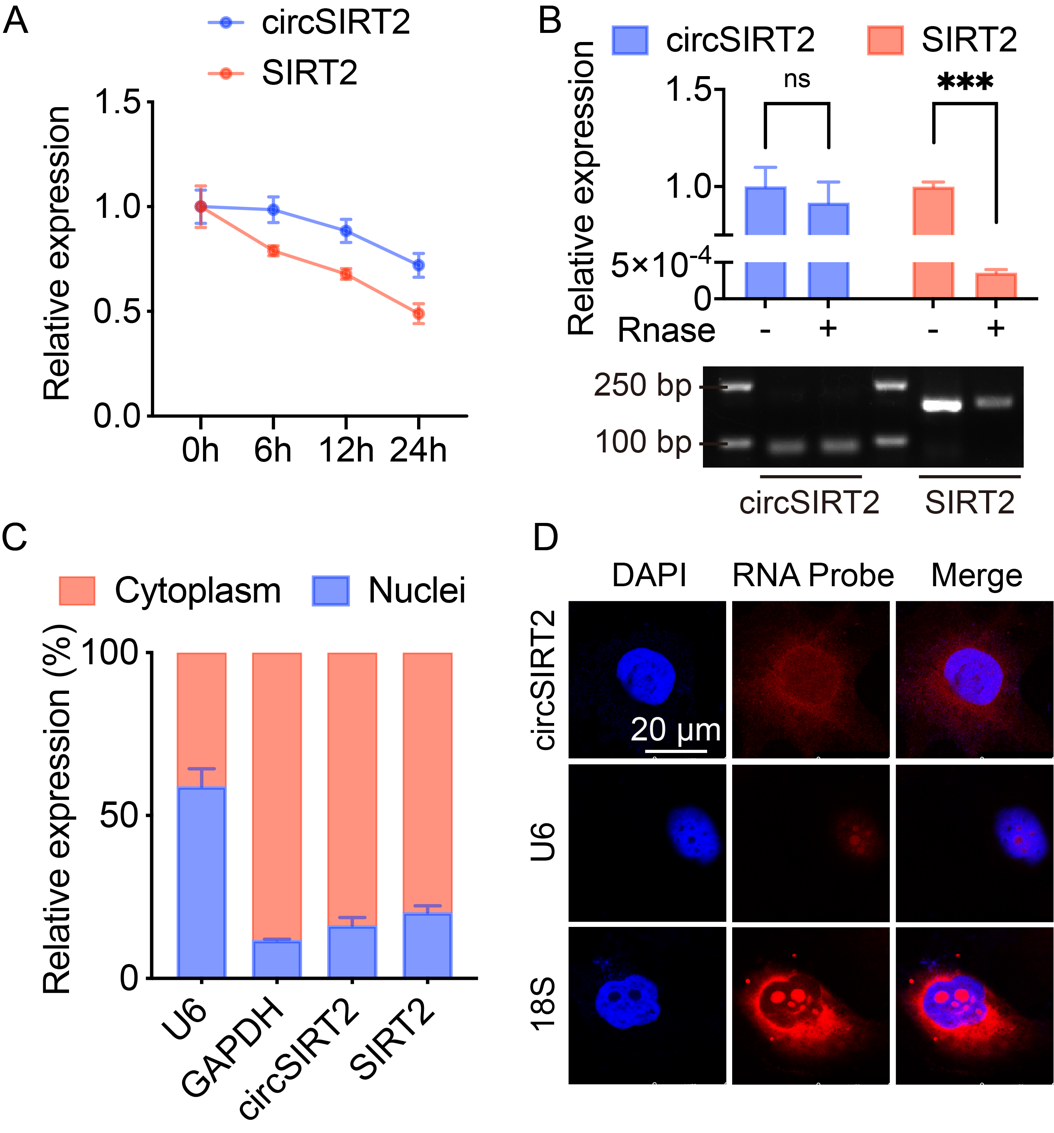


Supplementary Figure S4. The circRNA nature of circSIRT2.

1. Actinomycin D (ActD) treatment of circular and linear SIRT2 for different durations. ActD treatment inhibited transcription and RNA levels were verified by RT-qPCR. Results showed that the degradation of linear SIRT2 kept faster than that of circSIRT2 at each time point. n = 3 for each group at each time point.
2. Ribonuclease R (RNase R) treatment of circular and linear SIRT2. The relative levels of circular and linear SIRT2 with and without RNase R treatment were quantified by RT-qPCR. Results showed that circSIRT2 was resistant to the RNase R digestion. The PCR product size of circular and linear SIRT2 were 79 base pairs (bp) and 184 bp, respectively, as shown by the gel. n = 3 for each group.
3. Nucleocytoplasmic separation of circular and linear SIRT2. RT-qPCR was used to quantified their relative expression levels in nuclei and plasma. The expression levels of GAPDH and U6 were tested to verified the successful separation of nuclei and plasma. n = 3 for each group.
4. RNA- fluorescence in situ hybridization assay was performed to detect the expression patterns of circSIRT2. U6 and 18S were used as references. Nuclei were stained with DAPI.

Con = control, SRF = subretinal fibrosis, ns = not significant, ^*^*P* <0.05, ^**^*P* <0.01, ^***^*P* <0.001, independent two-sample student’s *t* test. Error bars indicated SEM.


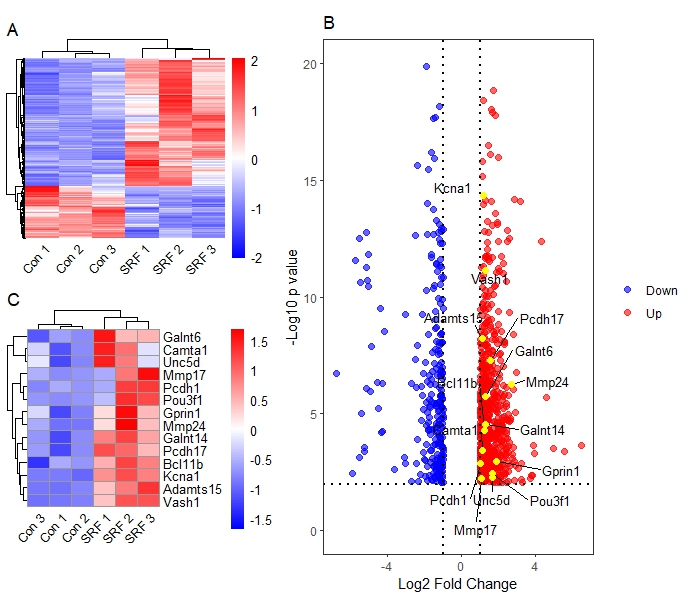


Supplementary Figure S5. Expression signatures in mouse subretinal fibrosis (SRF) models.

1. Whole-transcriptome sequencing of the retinal pigmental epithelium-choroid-sclera complexes of the mouse SRF models, visualized by R software.
2. Volcano plot of differentially expressed genes with absolute fold change >2 in mouse SRF models. The upregulated genes were shown in red and downregulated genes were shown in blue. Potential miR-542-3p target genes were highlighted with yellow dots.
3. Heatmap of miR-542-3p target gene candidates.

Con = control, SRF = subretinal fibrosis.

| Supplementary Table S1. small interfering RNA (siRNA) sequences used in this study. | |
| --- | --- |
| siRNA | Target sequence |
| si-h-hsa_circ_0050951_001 | CTGCTACACGCAGGTCGCA |
| si-h-hsa_circ_0050951_002 | ACGCAGGTCGCAGAGTCAT |
| si-h-hsa_circ_0050951_003 | GCAGGTCGCAGAGTCATCT |
| si-h-hsa_circ_0050951_004 (si-circSIRT2) | GCTACACGCAGGTCGCAGA |
| si-h-hsa_circ_0050951_005 | TACACGCAGGTCGCAGAGT |
| si-h-hsa_circ_0050951_006 | CACGCAGGTCGCAGAGTCA |
| genOFFTM st-h-VASH1 (si-VASH1) | CCATCAGCTTCAAGACCTA |

| Supplementary Table S2. Primers used in RT-qPCR. | | |
| --- | --- | --- |
|  | Forward primer | Reverse primer |
| GAPDH | CATGAGAAGTATGACAACAGCCT | AGTCCTTCCACGATACCAAAGT |
| m_circRNA_1593 | CCACACGTTTGAGGGTCTTG | TGGCTTTCCCTCTTACACTTGA |
| m_circRNA_3550 | TCGTAGTATTCATCACTTGCTCTGA | AAGACGGCCATCAAGAGACC |
| m_circRNA_6863 | AAACTGAAGGTAGCCGAGGG | TGGTTCAAGATCTTCACAACAGG |
| m_circRNA_3042 | CAGGAGCACGTCATCAGGTT | GCATCGTTCTTTGATGTCTCGG |
| m_circRNA_6794 | GCAGTCGAGCCCGTATTTCA | GGTGGAACTCATCCTGTCCG |
| m_circRNA_304 | ACCCCATCTCCATGACTACCT | AATGGTACCTTCCCTCCTGGT |
| m_circRNA_2745 | GCCTGAGGCCTGAAGACAAT | ATGAAGAGCTGCAGGCCAAT |
| m_circRNA_3613 | TGTGATGGTACCCAGCGAAC | ACCTTAAAAGGGCAATGCTGC |
| m_circRNA_5375 | CCGCATCCGGAAAACCATTC | CGGGAGGAGATCCTTTTGGG |
| m_circRNA_1969 | GCCTGGTGTGGCATATAGGT | ACCACATCGTCCACATAATTAAACT |
| m_circRNA_6228 (circSirt2) | TACACGCAGGCCGCAAG | GGAGCGGAAGTCAGGGATAC |
| circSIRT2 | CTACACGCAGGTCGCAGAGT | GAGAGCGAAAGTCGGGGATG |
| SIRT2 | CAGACTCTGAGGGAGGAGCC | CCAGCTCCCACCAAACAGAT |
| COL1A1 | GAGGGCCAAGACGAAGACATC | CAGATCACGTCATCGCACAAC |
| ACTA2 | GTGTTGCCCCTGAAGAGCAT | GCTGGGACATTGAAAGTCTCA |
| VIM | AGTCCACTGAGTACCGGAGAC | CATTTCACGCATCTGGCGTTC |
| miR-214-3p | GCAGGCACAGACAGGCAG | / |
| miR-542-3p | GCTGTGACAGATTGATAACTGAAA | / |
| miR-761 | GCAGCAGGGTGAAACTGACA | / |
| GPRIN1 | ATTCCAGGCTCATCAGGCAAG | GAAGCCGTAGTATCCGTGTGG |
| KCNA1 | TAGTGCAGTGTACTTTGCCGA | GTCACCGTATCCTACAGTGGT |
| PCDH17 | GCACGGTGATCGGGAACAT | GCGCTGCTTGGTGTAGAGG |
| CCL22 | ATCGCCTACAGACTGCACTC | GACGGTAACGGACGTAATCAC |
| CNNM1 | CTTCGCCACTGTCTCCGAG | GTCCAGTCGGGTGTCATTGAA |
| GALNT14 | CACTGCTGGTGTATTGCACG | CGGATCAGATGCGTAGGGG |
| GALNT6 | ACAGCGTCCTACACACCAC | CTTCTCCTTTAGGTGCTCCTCT |
| MMP24 | CCACTTTGACTCCGATGAGCC | TTGTGCGTCTCCATGTACTGG |
| PCDH1 | AACGGTGTGGCATCCTATGAG | GTTGCCCATCACAATGAGCTG |
| PSD4 | CAGGCAGAACACAGCATCAC | GGTCTAGGACACACATCTGGG |
| UNC5D | CAAGAGCAACCCTATTGCACT | CTCGTTCTGATGGACCCACTC |
| BCL11B | GGTGCCTGCTATGACAAGG | GGCTCGGACACTTTCCTGAG |
| CAMTA1 | TGCCGAAAACAAGCCGGAA | GGCAGACATTCAAGCAGCTTT |
| VASH1 | GGTGGGCTACCTGTGGATG | CACTCGGTATGGGGATCTTGG |
| ADAMTS15 | CCTGGACCCTTACAAGCCG | GGAACTTGACCATTGACTCGTC |
| MMP17 | CACTCATGTACTACGCCCTCA | TGGAGAAGTCGATCTGGATGTC |
| NYX | TTCCTTTTCCGCAATCCTTGG | GGACGTGGTAAAGTTCAGTTCA |
| CCL22 | ATCGCCTACAGACTGCACTC | GACGGTAACGGACGTAATCAC |
| POU3F1 | TACCGCGAAGTGCAGAAG | CGTGGGTAGCCACTGGGGG |
| Gapdh | AGGTCGGTGTGAACGGATTTG | TGTAGACCATGTAGTTGAGGTCA |
| Col1a1 | GCTCCTCTTAGGGGCCACT | CCACGTCTCACCATTGGGG |
| Acta2 | GTCCCAGACATCAGGGAGTAA | TCGGATACTTCAGCGTCAGGA |
| Vim | CGTCCACACGCACCTACAG | GGGGGATGAGGAATAGAGGCT |
| Unc5d | TGGCTAGGACTCTTTTTCTGGG | GCTCCTCGATGAAATGAGGCA |
| Vash1 | ACATGCGGCTCAAGATTGG | CTGTGGGGAGGAAACATCCTT |

| Supplementary Table S3. Sequences cloned into the pGL6-miR plasmid vector for the dual-luciferase reporter assay. | |
| --- | --- |
|  | Sequences |
| Dual-luciferase reporter assay for circSIRT2 and miR-542-3p or miR-214-3p | |
| LUC-circSIRT2-WT | GGTACC–GGGCAGTTCAAGCCAACCATCTGTCACTACTTCATGCGCCTGCTGAAGGA-AAGCTT |
| LUC-circSIRT2-MUT1 | GGTACC-GGGCATTGCAAGCCAACCATCTGTCACTACTTCATGCGCCTGCTGAAGGA-AAGCTT |
| LUC-circSIRT2-MUT2 | GGTACC-GGGCAGTTCAAGCCAACCATAGTGACATACTTCATGCGCCTGCTGAAGGA-AAGCTT |
| LUC-circSIRT2-MUT3 | GGTACC-GGGCAGTTCAAGCCAACCATCTGTCACTACTTCATGCGAAGTAGTAAGGA-AAGCTT |
| Dual-luciferase reporter assay for Vash1 mRNA 3’-UTR and miR-542-3p | |
| LUC-Vash1-WT | GGTACC-GCATCTCCAGCCAGGTGGGGCTGTCACTGTACTAG-AAGCTT |
| LUC-Vash1-MUT | GGTACC-GCATCTCCAGCCAGGTGGGGACTGACATGTACTAG-AAGCTT |
| WT = wild type, MUT = mutant. | |
